# Crystal structure of 4,6-dimethyl-2-{[3,4,5-trihy­droxy-6-(hy­droxy­meth­yl)tetra­hydro-2*H*-pyran-2-yl]sulfan­yl}nicotino­nitrile

**DOI:** 10.1107/S2056989017015213

**Published:** 2017-10-20

**Authors:** Doaa M. Masoud, Sherif F. Hammad, Galal H. Elgemeie, Peter G. Jones

**Affiliations:** a Chemistry Department, Faculty of Science, Helwan University, Cairo, Egypt; b Pharmaceutical Chemistry Department, Faculty of Pharmacy, Helwan University, Cairo, Egypt; c Institut für Anorganische und Analytische Chemie, Technische Universität Braunschweig, Hagenring 30, D-38106 Braunschweig, Germany

**Keywords:** crystal structure, pyridine, thio­glucose

## Abstract

In the title compound, C_14_H_18_N_2_O_5_S, the C—S bond lengths are unequal, with S—C_glucose_ = 1.8016 (15) Å and S—C_pyrid­yl_ = 1.7723 (13) Å. The hydro­philic glucose residues lie in the regions *z* ≃ 0.25 and 0.75. Four classical hydrogen bonds link the mol­ecules to form layers parallel to the *ab* plane, from which the pyridyl rings project; pyridyl ring stacking parallel to the *a* axis links adjacent layers.

## Abstract 2

In the title compound, the hydro­philic glucose residues lie in the regions *z* ≃ *ab* plane, from which the pyridyl rings project; pyridyl ring stacking parallel to the *a* axis links adjacent layers.

## Chemical context   

The search for new anti­cancer chemotherapeutic agents continues to be an active area of research (Elgemeie, 2003 ▸; Elgemeie & Jones, 2004 ▸). In recent years nucleoside analogs have occupied a significant position in the search for effective chemotherapeutic agents, because many non-natural nucleoside derivatives have been shown to possess bioactivity (Elgemeie & Abou-Zeid, 2015 ▸). In the last few decades, pyridine derivatives have received considerable attention because of their wide-ranging applications as anti­metabolic agents (Elgemeie *et al.*, 2009 ▸). Recently, we reported that many pyridine thio­glycosides showed strong cytotoxicity against several human cancer cell lines and block proliferation of various cancer cell lines (Elgemeie, Abou-Zeid *et al.*, 2015 ▸). We also showed that thio­glycosides involving pyridine and di­hydro­pyridine groups exerted inhibitory effects on both DNA- and RNA-containing viruses and inhibitors of protein glycosyl­ation, respectively (Elgemeie *et al.*, 2010 ▸). In view of these observations and with the aim of identifying new anti­cancer agents with improved pharmacokinetic and safety profiles, we have synthesized some new non-classical nucleoside analogs incorporating pyridine thio­glycosides.

We report here a novel one-step synthesis of a pyridine-2-thio­glucoside derivative by reaction of the pyridine-2(1*H*)-thione derivative (**1**) with 2,3,4,6-tetra-*O*-acetyl-α-d-gluco­pyranosyl bromide (**2**). Thus, (**1**) reacted with (**2**) in KOH/acetone to give a product for which two isomeric structures, (**3**) and (**4**), seemed possible, corresponding to two possible modes of glu­cosyl­ation. After deprotection of the product (see Scheme), the final free sugar pyridine­thione *N*-glucoside (**5**) or its regioisomer pyridine-2-thio­glucoside (**6**) was obtained. Spectroscopic data cannot differentiate between these structures.
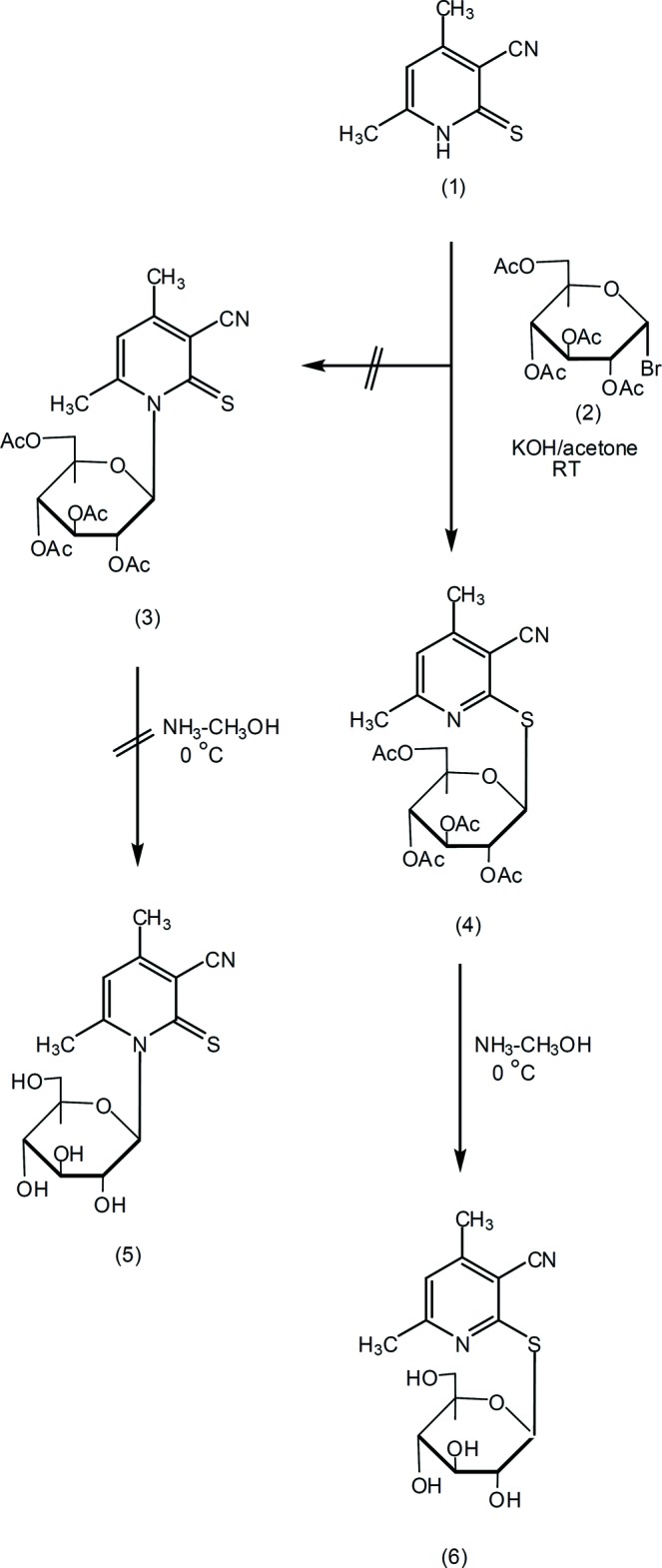


## Structural commentary   

**Figure 1:**
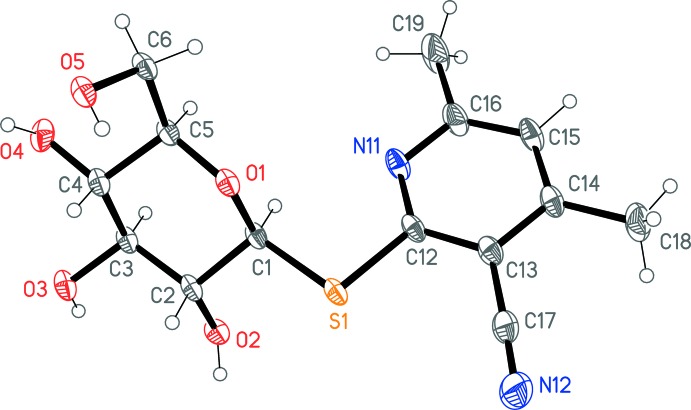
The structure of the title compound in the crystal. Displacement ellipsoids represent 50% probability levels.

The X-ray structure determination indicated unambiguously the formation of the pyridine-2-thio­glucoside (**6**) as the product in the solid state. The mol­ecule is shown in Fig. 1 ▸ and geometrical parameters are given in Table 1 ▸. The C—S bond lengths are markedly unequal, with S—C_glucose_ 1.8016 (15), S—C_pyrid­yl_ 1.7723 (13) Å. The main torsional degrees of freedom are between the rings, as defined by the torsion angles N11—C12—S1—C1 = −2.08 (14) and C2—C1—S1—C12 = 152.98 (9)°.

## Supra­molecular features   

**Figure 2:**
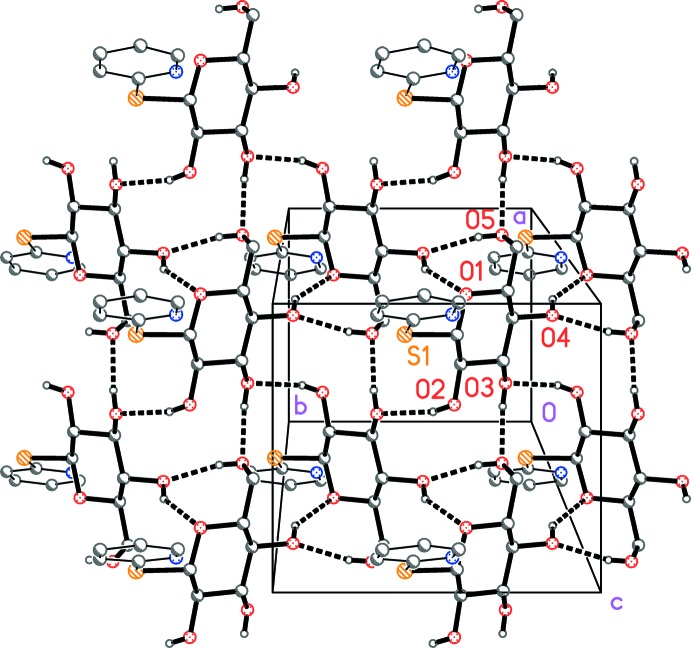
Packing diagram of the title compound, viewed perpendicular to the *ab* plane. Classical hydrogen bonds are indicated by dashed lines. Methyl and nitrile substituents of the pyridine rings have been omitted for clarity.

The glucose moieties of (**6**) occupy the regions at *z* ≃ 0.25 and 0.75. The layer structure of (**6**) is shown in Fig. 2 ▸. Each O—H donor forms one two-centre hydrogen bond (Table 2 ▸); the two most linear C—H⋯*X* inter­actions also lie within the layer, but are not drawn explicitly in Fig. 2 ▸. The same applies to the short contact S1⋯O5 (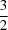 − *x*, 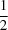 + *y*, 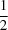 − *z*) = 3.1417 (10) Å.

Adjacent layers are connected *via* the pyridyl rings, which project into the spaces between the hydro­philic layers and form π stacks parallel to the *a* axis. Adjacent rings in the stack are related by the twofold axis (operators 1 − *x*, *y*, 1 − *z* and 2 − *x*, *y*, 1 − *z*). The inter­planar angles are 4.33 (5)°, the centroid-to-centroid distances are 3.96 and 3.72 Å, and the ring offsets are *ca* 1.26 and 0.94 Å; these cannot be expressed exactly because neighbouring rings are not exactly parallel.

## Database survey   

Perhaps surprisingly, a database search revealed only one other example of a pyridine ring with a thio­glucose substituent at the 2-position, namely pyridyl thio­glucose monohydrate (Nordenson & Jeffrey, 1980 ▸; refcode PYSGPR). This compound also shows a marked inequality between the S—C bond lengths (*cf*. Table 1 ▸); S—C_glucose_ is 1.793 (3), S—C_pyrid­yl_ is 1.759 (3) Å.

## Synthesis and crystallization   

To a solution of the pyridine-2-(1*H*)-thione (**1**) (1.64 gm, 0.01 mol) in aqueous potassium hydroxide (6 ml, 0.56 g, 0.01 mol) was added a solution of 2,3,4,6-tetra-*O*-acetyl-α-d-gluco­pyranosyl bromide (**2**) (4.52 g, 0.011 mol) in acetone (30 ml). The reaction mixture was stirred at room temperature until the reaction was judged complete by TLC (30 min to 2 h). The mixture was evaporated under reduced pressure at 313 K and the residue was washed with distilled water to remove the potassium bromide. The solid was collected by filtration and crystallized from ethanol to give compound (**3**) in 85% yield (m.p. 468 K). Dry gaseous ammonia was then passed through a solution of the protected thio­glycoside (**3**) (0.5 g) in dry methanol (20 ml) at 273 K for 0.5 h, then the mixture was stirred at 273 K until completion of the reaction (TLC, 2–6 h). The mixture was evaporated at 313 K to give a solid residue, which was recrystallized from ethanol to give compound (**6**) in 85% yield (m.p. 482–483 K).

IR (KBr): 3600–3258 (OH), 2222 (CN) cm^−1^. ^1^H NMR (DMSO-*d*_6_): δ 2.22 (*s*, 3H, CH_3_), 2.31 (*s*, 3H, CH_3_), 3.15–3.80 (*m*, 6H, 2H-6′, H-5′, H-4′, H-3′, H-2′), 4.40 (*d*, *J* = 9.55 Hz, 2H, HO-2′ and HO-3′), 4.90 (*s*, 1H, HO-4′), 5.30 (*s*, 1H, HO-6′), 5.59 (*d*, *J*_1,2_ = 9.86 Hz, 1H, H-1′), 7.19 (*s*, 1H, pyridine H-5) ppm. ^13^C NMR: δ 20.7 (CH_3_), 22.9 (CH_3_), 61.0 (C6′), 68.9 (C4′), 72.7 (C2′), 75.9 (C3′), 80.7 (C5′), 83.7 (C1′), 103.2 (C3), 116.0 (CN), 119.7 (C5), 149.2 (C4), 159.0 (C6), 164.0 (C2) ppm.

## Refinement   

Crystal data, data collection and structure refinement details are summarized in Table 3 ▸. The space group as initially found by the diffractometer program was *P*2_1_, with two independent but virtually identical mol­ecules in the asymmetric unit. It became apparent that the two mol­ecules formed layer structures independent of each other, and were related by a translation vector (0.5, 0.5, 0.5). The *checkCIF* program also indicated that the true space group should be centred, with a 100% fit and a small deviation. The same cell was retained for ease of checking, and the structure determination and refinement repeated in space group *I*2. The refinement was entirely satisfactory, and corresponds to the structure presented here. However, the reflections with (*h* + *k* + *l*) odd, which are required to be systematically absent in *I*2, seemed to be quite definitely present. We are unable to explain this anomaly. The HKL file appended to the CIF contains these reflections.

Crystal data, data collection and structure refinement details are summarized in Table 2 ▸. OH hydrogen atoms were refined freely but with an O—H distance restraint (SADI). Methyl groups were refined as idealized rigid groups allowed to rotate but not tip (AFIX 137), with C—H 0.98 Å and H—C—H 109.5°. Other hydrogen atoms were included using a riding model starting from calculated positions (C—H_aromatic_ 0.95, C—H_methyl­ene_ 0.99, C—H_methine_ 1.00 Å).

The absolute configuration was determined by the unambiguous Flack parameter of −0.008 (8) (Parsons *et al.*, 2013 ▸).

## Supplementary Material

Crystal structure: contains datablock(s) I, global. DOI: 10.1107/S2056989017015213/qm2120sup1.cif

Structure factors: contains datablock(s) I. DOI: 10.1107/S2056989017015213/qm2120Isup2.hkl

CCDC reference: 1580696

Additional supporting information:  crystallographic information; 3D view; checkCIF report

## Figures and Tables

**Table 1:** Selected geometric parameters (Å, °)

S1—C12	1.7723 (13)	S1—C1	1.8016 (15)
			
C12—S1—C1	100.43 (6)		
			
C12—S1—C1—C2	152.98 (9)	C1—S1—C12—N11	−2.08 (14)

**Table 2:** Hydrogen-bond geometry (Å, °)

*D*—H⋯*A*	*D*—H	H⋯*A*	*D*⋯*A*	*D*—H⋯*A*
O2—H02⋯O3^i^	0.80 (2)	2.01 (2)	2.7909 (16)	165 (3)
O3—H03⋯O5^ii^	0.81 (2)	1.93 (2)	2.7394 (14)	174 (2)
O4—H04⋯O1^iii^	0.80 (2)	2.06 (2)	2.7490 (15)	144 (2)
O5—H05⋯O4^iv^	0.79 (2)	1.96 (2)	2.7324 (18)	165 (3)
C2—H2⋯O5^iv^	1.00	2.47	3.3326 (19)	144
C5—H5⋯N12^v^	1.00	2.64	3.638 (2)	179

Symmetry codes: (i) 
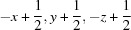
; (ii) 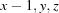; (iii) 
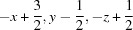
; (iv) 
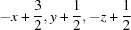
; (v) 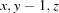.

**Table 3:** Experimental details

Crystal data	
Chemical formula	C_14_H_18_N_2_O_5_S
*M*_r_	326.36
Crystal system, space group	Monoclinic, *I*2
Temperature (K)	100
*a*, *b*, *c* (Å)	7.66978 (18), 8.72860 (13), 23.7524 (4)
β (°)	98.7356 (16)
*V* (Å^3^)	1571.69 (5)
*Z*	4
Radiation type	Mo *K*α
μ (mm^−1^)	0.23
Crystal size (mm)	0.40 × 0.35 × 0.20

Data collection	
Diffractometer	Oxford Diffraction Xcalibur Eos
Absorption correction	Multi-scan (*CrysAlis PRO*; Rigaku Oxford Diffraction, 2015 ▸)
*T*_min_, *T*_max_	0.980, 1.000
No. of measured, independent and observed [*I* > 2σ(*I*)] reflections	84844, 4741, 4590
*R*_int_	0.027
(sin θ/λ)_max_ (Å^−1^)	0.729

Refinement	
*R*[*F* ^2^ > 2σ(*F* ^2^)], *wR*(*F* ^2^), *S*	0.024, 0.063, 1.05
No. of reflections	4741
No. of parameters	217
No. of restraints	7
H-atom treatment	H atoms treated by a mixture of independent and constrained refinement
Δρ_max_, Δρ_min_ (e Å^−3^)	0.30, −0.17
Absolute structure	Flack *x* determined using 2066 quotients [(*I* ^+^)−(*I* ^−^)]/[(*I* ^+^)+(*I* ^−^)] (Parsons *et al.*, 2013 ▸)
Absolute structure parameter	−0.008 (8)

Computer programs: *CrysAlis PRO* (Rigaku Oxford Diffraction, 2015 ▸), *SHELXS97* (Sheldrick, 2008 ▸), *SHELXL2017* (Sheldrick, 2015 ▸) and *XP* (Siemens, 1994 ▸).

## supplementary crystallographic information

### Crystal data

**Table:**

C_14_H_18_N_2_O_5_S	*F*(000) = 688
*M**_r_* = 326.36	*D*_x_ = 1.379 Mg m^−^^3^
Monoclinic, *I*2	Mo *K*α radiation, λ = 0.71073 Å
*a* = 7.66978 (18) Å	Cell parameters from 46975 reflections
*b* = 8.72860 (13) Å	θ = 2.7–30.9°
*c* = 23.7524 (4) Å	µ = 0.23 mm^−^^1^
β = 98.7356 (16)°	*T* = 100 K
*V* = 1571.69 (5) Å^3^	Block, colourless
*Z* = 4	0.40 × 0.35 × 0.20 mm

### Data collection

**Table:**

Oxford Diffraction Xcalibur Eos diffractometer	4741 independent reflections
Radiation source: Enhance (Mo) X-ray Source	4590 reflections with *I* > 2σ(*I*)
Detector resolution: 16.1419 pixels mm^-1^	*R*_int_ = 0.027
ω–scan	θ_max_ = 31.2°, θ_min_ = 2.5°
Absorption correction: multi-scan (*CrysAlis PRO*; Rigaku Oxford Diffraction, 2015)	*h* = −10→11
*T*_min_ = 0.980, *T*_max_ = 1.000	*k* = −12→12
84844 measured reflections	*l* = −34→33

### Refinement

**Table:**

Refinement on *F*^2^	Hydrogen site location: mixed
Least-squares matrix: full	H atoms treated by a mixture of independent and constrained refinement
*R*[*F*^2^ > 2σ(*F*^2^)] = 0.024	*w* = 1/[σ^2^(*F*_o_^2^) + (0.0354*P*)^2^ + 0.4674*P*] where *P* = (*F*_o_^2^ + 2*F*_c_^2^)/3
*wR*(*F*^2^) = 0.063	(Δ/σ)_max_ = 0.001
*S* = 1.05	Δρ_max_ = 0.30 e Å^−^^3^
4741 reflections	Δρ_min_ = −0.17 e Å^−^^3^
217 parameters	Absolute structure: Flack *x* determined using 2066 quotients [(*I*^+^)-(*I*^-^)]/[(*I*^+^)+(*I*^-^)] (Parsons *et al.*, 2013)
7 restraints	Absolute structure parameter: −0.008 (8)

### Special details

**Table:**

Geometry. All esds (except the esd in the dihedral angle between two l.s. planes) are estimated using the full covariance matrix. The cell esds are taken into account individually in the estimation of esds in distances, angles and torsion angles; correlations between esds in cell parameters are only used when they are defined by crystal symmetry. An approximate (isotropic) treatment of cell esds is used for estimating esds involving l.s. planes.

### Fractional atomic coordinates and isotropic or equivalent isotropic displacement parameters (Å^2^)

**Table:**

	*x*	*y*	*z*	*U*_iso_*/*U*_eq_	
S1	0.59062 (4)	0.54939 (4)	0.37504 (2)	0.01953 (8)	
C1	0.57772 (17)	0.35296 (17)	0.35150 (5)	0.0175 (2)	
H1	0.557606	0.284848	0.383749	0.021*	
O1	0.74310 (12)	0.31545 (12)	0.33376 (4)	0.0183 (2)	
C2	0.42750 (17)	0.33167 (17)	0.30152 (5)	0.0177 (3)	
H2	0.444291	0.404110	0.270164	0.021*	
O2	0.26172 (13)	0.35885 (13)	0.31878 (4)	0.0200 (2)	
H02	0.223 (4)	0.438 (3)	0.3050 (12)	0.054 (8)*	
C3	0.42986 (17)	0.16706 (17)	0.27975 (5)	0.0184 (2)	
H3	0.399828	0.095244	0.309645	0.022*	
O3	0.30583 (13)	0.14881 (14)	0.22910 (4)	0.0226 (2)	
H03	0.207 (3)	0.161 (3)	0.2365 (9)	0.033 (6)*	
C4	0.61090 (18)	0.12809 (18)	0.26570 (6)	0.0185 (3)	
H4	0.636616	0.194695	0.233704	0.022*	
O4	0.61422 (15)	−0.02842 (14)	0.24885 (5)	0.0250 (2)	
H04	0.672 (3)	−0.035 (3)	0.2237 (9)	0.039 (6)*	
C5	0.74991 (17)	0.15622 (18)	0.31794 (5)	0.0186 (2)	
H5	0.722628	0.091101	0.350129	0.022*	
C6	0.93568 (18)	0.12113 (19)	0.30688 (5)	0.0210 (3)	
H6A	1.021081	0.174340	0.335809	0.025*	
H6B	0.957132	0.009636	0.311368	0.025*	
O5	0.96641 (13)	0.16663 (14)	0.25148 (4)	0.0208 (2)	
H05	0.957 (3)	0.256 (2)	0.2480 (11)	0.043 (7)*	
N11	0.71744 (19)	0.38506 (16)	0.46739 (5)	0.0247 (3)	
C12	0.68518 (18)	0.52490 (16)	0.44732 (5)	0.0193 (3)	
C13	0.7223 (2)	0.65733 (19)	0.48057 (6)	0.0219 (3)	
C14	0.8011 (2)	0.64238 (19)	0.53789 (6)	0.0235 (3)	
C15	0.8355 (2)	0.4955 (2)	0.55786 (6)	0.0274 (3)	
H15	0.889755	0.479840	0.596114	0.033*	
C16	0.7915 (2)	0.3696 (2)	0.52236 (6)	0.0290 (3)	
C17	0.6799 (3)	0.8053 (2)	0.45591 (7)	0.0313 (4)	
N12	0.6448 (3)	0.9219 (2)	0.43492 (7)	0.0491 (5)	
C18	0.8435 (3)	0.7795 (2)	0.57556 (7)	0.0357 (4)	
H18A	0.876528	0.745852	0.615104	0.054*	
H18B	0.739865	0.846339	0.572717	0.054*	
H18C	0.941903	0.835962	0.563468	0.054*	
C19	0.8230 (4)	0.2094 (2)	0.54374 (8)	0.0472 (5)	
H19A	0.779315	0.137031	0.513317	0.071*	
H19B	0.760624	0.192842	0.576301	0.071*	
H19C	0.949717	0.193415	0.555621	0.071*	

### Atomic displacement parameters (Å^2^)

**Table:**

	*U*^11^	*U*^22^	*U*^33^	*U*^12^	*U*^13^	*U*^23^
S1	0.01731 (15)	0.02917 (15)	0.01144 (12)	−0.00026 (13)	0.00001 (10)	0.00067 (12)
C1	0.0108 (6)	0.0311 (7)	0.0110 (5)	−0.0011 (5)	0.0029 (4)	−0.0017 (5)
O1	0.0094 (4)	0.0321 (5)	0.0138 (4)	−0.0017 (4)	0.0028 (3)	−0.0016 (4)
C2	0.0091 (6)	0.0334 (7)	0.0107 (5)	−0.0015 (5)	0.0023 (4)	−0.0006 (5)
O2	0.0097 (4)	0.0329 (5)	0.0181 (4)	0.0011 (4)	0.0045 (3)	0.0002 (4)
C3	0.0094 (6)	0.0328 (7)	0.0132 (5)	−0.0023 (5)	0.0029 (4)	−0.0023 (5)
O3	0.0084 (4)	0.0429 (6)	0.0162 (4)	−0.0026 (4)	0.0014 (3)	−0.0066 (4)
C4	0.0093 (6)	0.0329 (7)	0.0136 (5)	−0.0026 (5)	0.0029 (4)	−0.0033 (5)
O4	0.0159 (5)	0.0371 (6)	0.0239 (5)	−0.0036 (4)	0.0095 (4)	−0.0100 (5)
C5	0.0114 (6)	0.0322 (6)	0.0123 (5)	−0.0006 (5)	0.0025 (4)	−0.0015 (5)
C6	0.0104 (6)	0.0404 (8)	0.0124 (5)	0.0011 (5)	0.0018 (4)	−0.0012 (5)
O5	0.0113 (4)	0.0378 (6)	0.0138 (4)	−0.0013 (4)	0.0040 (3)	−0.0025 (4)
N11	0.0303 (7)	0.0304 (6)	0.0123 (5)	−0.0068 (5)	−0.0003 (5)	0.0028 (5)
C12	0.0153 (6)	0.0318 (8)	0.0107 (5)	−0.0027 (5)	0.0022 (4)	0.0005 (5)
C13	0.0213 (7)	0.0316 (7)	0.0131 (6)	−0.0011 (6)	0.0039 (5)	−0.0001 (5)
C14	0.0249 (7)	0.0330 (8)	0.0127 (6)	−0.0042 (6)	0.0030 (5)	−0.0021 (5)
C15	0.0331 (8)	0.0353 (7)	0.0124 (6)	−0.0069 (6)	−0.0010 (5)	0.0010 (5)
C16	0.0397 (9)	0.0319 (8)	0.0139 (6)	−0.0065 (7)	−0.0009 (6)	0.0042 (6)
C17	0.0441 (10)	0.0340 (8)	0.0151 (6)	0.0055 (7)	0.0029 (6)	−0.0042 (6)
N12	0.0849 (15)	0.0372 (9)	0.0232 (7)	0.0143 (9)	0.0022 (8)	−0.0021 (6)
C18	0.0511 (11)	0.0371 (9)	0.0175 (7)	−0.0039 (8)	0.0005 (7)	−0.0059 (6)
C19	0.0847 (17)	0.0335 (9)	0.0182 (7)	−0.0065 (10)	−0.0093 (8)	0.0069 (7)

### Geometric parameters (Å, º)

**Table:**

S1—C12	1.7723 (13)	C6—H6A	0.9900
S1—C1	1.8016 (15)	C6—H6B	0.9900
C1—O1	1.4338 (16)	O5—H05	0.79 (2)
C1—C2	1.5348 (17)	N11—C12	1.3201 (19)
C1—H1	1.0000	N11—C16	1.3492 (19)
O1—C5	1.4428 (18)	C12—C13	1.405 (2)
C2—O2	1.4141 (16)	C13—C14	1.4092 (19)
C2—C3	1.528 (2)	C13—C17	1.435 (2)
C2—H2	1.0000	C14—C15	1.379 (2)
O2—H02	0.80 (2)	C14—C18	1.500 (2)
C3—O3	1.4247 (16)	C15—C16	1.394 (2)
C3—C4	1.5152 (18)	C15—H15	0.9500
C3—H3	1.0000	C16—C19	1.495 (2)
O3—H03	0.809 (19)	C17—N12	1.147 (2)
C4—O4	1.4249 (18)	C18—H18A	0.9800
C4—C5	1.5280 (18)	C18—H18B	0.9800
C4—H4	1.0000	C18—H18C	0.9800
O4—H04	0.800 (19)	C19—H19A	0.9800
C5—C6	1.5187 (18)	C19—H19B	0.9800
C5—H5	1.0000	C19—H19C	0.9800
C6—O5	1.4279 (16)		
			
C12—S1—C1	100.43 (6)	O5—C6—H6A	108.9
O1—C1—C2	109.80 (10)	C5—C6—H6A	108.9
O1—C1—S1	107.40 (9)	O5—C6—H6B	108.9
C2—C1—S1	110.77 (10)	C5—C6—H6B	108.9
O1—C1—H1	109.6	H6A—C6—H6B	107.8
C2—C1—H1	109.6	C6—O5—H05	110.3 (19)
S1—C1—H1	109.6	C12—N11—C16	118.04 (14)
C1—O1—C5	111.41 (10)	N11—C12—C13	123.16 (13)
O2—C2—C3	108.25 (11)	N11—C12—S1	119.22 (10)
O2—C2—C1	110.96 (10)	C13—C12—S1	117.62 (11)
C3—C2—C1	109.24 (11)	C12—C13—C14	119.18 (14)
O2—C2—H2	109.5	C12—C13—C17	119.82 (13)
C3—C2—H2	109.5	C14—C13—C17	121.00 (15)
C1—C2—H2	109.5	C15—C14—C13	116.75 (14)
C2—O2—H02	109 (2)	C15—C14—C18	121.60 (13)
O3—C3—C4	107.80 (10)	C13—C14—C18	121.64 (15)
O3—C3—C2	110.49 (11)	C14—C15—C16	120.56 (14)
C4—C3—C2	110.12 (11)	C14—C15—H15	119.7
O3—C3—H3	109.5	C16—C15—H15	119.7
C4—C3—H3	109.5	N11—C16—C15	122.29 (15)
C2—C3—H3	109.5	N11—C16—C19	116.40 (15)
C3—O3—H03	109.1 (16)	C15—C16—C19	121.31 (14)
O4—C4—C3	109.46 (12)	N12—C17—C13	178.31 (17)
O4—C4—C5	110.01 (12)	C14—C18—H18A	109.5
C3—C4—C5	109.57 (11)	C14—C18—H18B	109.5
O4—C4—H4	109.3	H18A—C18—H18B	109.5
C3—C4—H4	109.3	C14—C18—H18C	109.5
C5—C4—H4	109.3	H18A—C18—H18C	109.5
C4—O4—H04	108.6 (19)	H18B—C18—H18C	109.5
O1—C5—C6	108.14 (11)	C16—C19—H19A	109.5
O1—C5—C4	108.53 (11)	C16—C19—H19B	109.5
C6—C5—C4	112.61 (11)	H19A—C19—H19B	109.5
O1—C5—H5	109.2	C16—C19—H19C	109.5
C6—C5—H5	109.2	H19A—C19—H19C	109.5
C4—C5—H5	109.2	H19B—C19—H19C	109.5
O5—C6—C5	113.19 (11)		
			
C12—S1—C1—O1	−87.12 (9)	C3—C4—C5—C6	−179.39 (13)
C12—S1—C1—C2	152.98 (9)	O1—C5—C6—O5	−81.24 (14)
C2—C1—O1—C5	−63.63 (14)	C4—C5—C6—O5	38.67 (18)
S1—C1—O1—C5	175.85 (8)	C16—N11—C12—C13	−0.8 (2)
O1—C1—C2—O2	176.40 (11)	C16—N11—C12—S1	179.18 (12)
S1—C1—C2—O2	−65.14 (13)	C1—S1—C12—N11	−2.08 (14)
O1—C1—C2—C3	57.13 (14)	C1—S1—C12—C13	177.90 (11)
S1—C1—C2—C3	175.59 (8)	N11—C12—C13—C14	1.4 (2)
O2—C2—C3—O3	66.09 (13)	S1—C12—C13—C14	−178.62 (11)
C1—C2—C3—O3	−172.97 (10)	N11—C12—C13—C17	−178.71 (16)
O2—C2—C3—C4	−174.93 (11)	S1—C12—C13—C17	1.31 (19)
C1—C2—C3—C4	−54.00 (14)	C12—C13—C14—C15	−0.6 (2)
O3—C3—C4—O4	−62.97 (14)	C17—C13—C14—C15	179.51 (16)
C2—C3—C4—O4	176.42 (10)	C12—C13—C14—C18	−179.89 (15)
O3—C3—C4—C5	176.33 (12)	C17—C13—C14—C18	0.2 (2)
C2—C3—C4—C5	55.72 (15)	C13—C14—C15—C16	−0.7 (3)
C1—O1—C5—C6	−173.06 (10)	C18—C14—C15—C16	178.65 (17)
C1—O1—C5—C4	64.49 (13)	C12—N11—C16—C15	−0.5 (3)
O4—C4—C5—O1	179.92 (10)	C12—N11—C16—C19	178.95 (18)
C3—C4—C5—O1	−59.70 (14)	C14—C15—C16—N11	1.3 (3)
O4—C4—C5—C6	60.23 (16)	C14—C15—C16—C19	−178.17 (19)

### Hydrogen-bond geometry (Å, º)

**Table:**

*D*—H···*A*	*D*—H	H···*A*	*D*···*A*	*D*—H···*A*
O2—H02···O3^i^	0.80 (2)	2.01 (2)	2.7909 (16)	165 (3)
O3—H03···O5^ii^	0.81 (2)	1.93 (2)	2.7394 (14)	174 (2)
O4—H04···O1^iii^	0.80 (2)	2.06 (2)	2.7490 (15)	144 (2)
O5—H05···O4^iv^	0.79 (2)	1.96 (2)	2.7324 (18)	165 (3)
C2—H2···O5^iv^	1.00	2.47	3.3326 (19)	144
C5—H5···N12^v^	1.00	2.64	3.638 (2)	179

Symmetry codes: (i) −*x*+1/2, *y*+1/2, −*z*+1/2; (ii) *x*−1, *y*, *z*; (iii) −*x*+3/2, *y*−1/2, −*z*+1/2; (iv) −*x*+3/2, *y*+1/2, −*z*+1/2; (v) *x*, *y*−1, *z*.
